# Supplementary material for: Research on the impact mechanism of digital technology on the new-quality productivity of port logistics
Source: PLoS One. 2025 Sep 26;20(9):e0333071. doi: 10.1371/journal.pone.0333071 (PMC12469090; doi:10.1371/journal.pone.0333071)
Supplement: Attachment — Submitted filename: Response Letter.docx [file pone.0333071.s001.docx]

Response Letter

Dear Editor and Reviewers:

Thank you for your letter and for the reviewers’ comments concerning our manuscript entitled “Research on the Impact Mechanism of Digital Technology on the New-Quality Productivity of Port Logistics”(Manuscript ID: PONE-D-25-08375). Those comments are all valuable and very helpful for revising and improving our manuscript, as well as the important guiding significance to our research. We thank you very much for allowing us to revise our manuscript.

These feedbacks provide important guidelines for improving the quality of the article, and we have carefully sorted out and actively implemented the modifications, and now report the details as follows:

**Reviewer1:**

**Q1: The introduction lacks focus, innovation, and a concise structural overview.**

**Response:** We supplemented the limitations of existing research theories in the first paragraph of the introduction to clarify the research core of this paper: "First, the study mostly focuses on unidimensional improvements such as port transport efficiency, ignoring the systemic changes triggered by digital technologies; Second, it does not reveal the mediating role of digital infrastructure upgrades and the level of internalization of technology applications in the process of transformation of the new quality productivity”; the third paragraph is expanded on the basis of the original content to include a succinct overview of the structure, so that the logic of the introduction is more clear. (See lines 5 to 15 on page 3 and line 42 on page 3 to line 15 on page 4 for details)

**Q2: A structural equation model (SEM) diagram is needed to visually represent variable relationships and hypotheses. Figure 1 does not align with the hypotheses in later sections; it should be redesigned for consistency with the theoretical framework.**

**Response:** Thank you for valuable feedback. You pointed out the need for structural equation modeling diagrams to visualize the variable relationships and hypotheses, and pointed out the inconsistency between Figure 1 and the hypotheses, which is crucial for improving the presentation of the study. We have revised Figure 1 by adding two mediating variables in the new quality productivity of port logistics enabled by digital technology to make it consistent with the theoretical framework; meanwhile, we have added a new structural equation modeling diagram for the mechanism test analysis in Chapter 4.5, which presents in detail the composition of the evaluation indicators of digital technology and new quality productivity, as well as the influence coefficients among the explanatory variables, the explanatory variables and the mediating variables. (See lines 30 to 32 on page 8 and lines 8 to 9 on page 20 for details)

**Q3: “The regression models (4) and (5) purportedly validate H4”, but the hypothesis itself is not explicitly defined in the text. Ensure H4 is clearly stated and linked to these models.**

**Response:** Thank you for your suggestions. Regarding the issue of “H4 assumption is not clearly defined in the text”, we reorganize the correspondence between the model and the assumptions constructed in the text, and reset the model (1) to be linked to assumption H1, the model (2)(3) to be linked to assumption H2, and the model (4)(5) to be linked to assumption H3. This ensures the rigor of the article. (See lines 6 to 16 on page 14 for details)

**Q4: Expand the variable descriptions to include selection criteria and justification for their inclusion; a summary table is recommended. The explanation of Table 2 lacks coherence with its content. List specific foundational studies in section 3.2.3 “Referring to existing studies”.**

**Response:** Based on your suggestions, we have added variable descriptions and rationale for the selection of primary and secondary indicators before sections 3.2.1 and 3.2.2:” The first-level indicator adopts intangible labor data to reflect the intensity of the port's investment in intangible assets such as patents, software and brands, which is the core characterization of innovation-driven productivity; fixed labor data directly reflects the comprehensive processing capacity of the port logistics system, which also affects the process of automation and intelligent transformation; high-quality talents are the foundation of the human capital to promote the digital transformation and technological innovation; the financial risk indicator reflects the enterprise's investment The financial risk indicator reflects the enterprise's investment capacity, which affects the introduction of new technologies and facility upgrading; the future development indicator reflects the agility of the port logistics system and its ability to optimize the operation of the existing assets, which is highly compatible with the cost reduction and efficiency enhancement goals required by the new quality productivity.”; We added columns for indicator selection criteria in Tables 1 and 2; And we added references to existing studies in section 3.2.3 to make the variable-related content more detailed. (See lines 30 to 32 on page 8 and lines 8-9 on page 20 for details)

**Q5: Section 4.4.1: The exclusion of outlier years (2019–2020) to account for pandemic impacts is insufficient. Extend the period to 2020–2021, as port logistics were more severely disrupted during this time.**

**Response:** Thank you for your valuable proposal. We fully agree with your request to extend the period of excluded anomalous years to 2020 - 2021. The excluded anomalous years have been adjusted to 2020 - 2021 in Section 4.4.1 to more reasonably account for the shocks received by port logistics. (See lines 3 to 7 on page 17 for details)

**Q6: The dataset spans 2013–2022. Consider updating it to include the most recent data where feasible.**

**Response:** Thank you for your careful review and valuable suggestions on our manuscript. We re-collected data for the year 2023 and updated the full-text data to ensure that the study data is current. (See lines 19 to 23 on page 14 and line 1 on page 15 for details)

**Q7: The current instrumental variable selection and methodology for the endogeneity test lack empirical rigor. Reassess the approach using established econometric techniques to strengthen validity.**

**Response:** We are very grateful for your valuable proposal. In the endogeneity test, we re-selected the breadth of digital technology application as an instrumental variable, referring to Yang Peng's approach. Yang Peng used text analysis to search, match, and count the word frequency of keywords related to “digital technology” in the annual reports of listed companies according to the breadth of digital technology application, and expanded the sample in the year of study. We used established econometric techniques to re-evaluate the methodology and enhance the empirical rigor of the study. (See lines 4 to 24 on page 16 and line 1 on page 17 for details)

**Q8: A comparative experiment involving the three ports is considered for addition to test whether differences among ports affect the conclusion.**

**Response:** Thank you for your careful review and valuable suggestions. Your suggestion to add a comparative experiment involving three ports is of great academic value and practical guidance for exploring in depth how differences between ports affect research findings. Through the comparative experiment, the impact of the differences in geographic location, scale of operation, and policy environment on the relationship between digital technology and new quality productivity can be more clearly identified, thus making the research conclusions more generalizable and in-depth. However, due to the limitations of research time and data acquisition, we regret that we are unable to conduct this experiment for the time being. We will incorporate this into our research program as soon as conditions permit.

**Q9: Standardize citation formats throughout the manuscript and ensure consistency in reference list formatting.**

**Response:** We have overhauled the formatting of the bibliographic citations to ensure that the citation formatting and the formatting of the reference lists are consistent throughout the manuscript.” Burinskiene A, Daskevic D. The investigation on the application of digital technologies for logistics business competitiveness[J]. Tehnički glasnik, 2024, 18(4): 626-637.”. (See lines on page 23 to page 26 for details)

**Q10: Expand the discussion of Managerial insights, particularly regarding how port authorities and policymakers can leverage digital technologies to enhance new quality productivity. Provide actionable recommendations based on empirical findings.**

**Response:** Thank you for your valuable suggestions. In conjunction with the empirical results, we further expand the discussion of management insights by providing actionable recommendations for port authorities and policymakers at three levels. (See lines 15 to 44 on page 21 and lines 1 to 5 on page 22 for details)

Thank you again for your positive and constructive comments and suggestions on our manuscript.

**Reviewer2:**

**Q1: The literature review primarily discusses the relationship between digital technology and productivity. However, it provides limited discussion on the specific measurement indicators of new-quality productivity. It is recommended to supplement relevant theoretical discussions to strengthen the conceptual foundation.**

**Response:** Thank you for your valuable proposal. Your suggestion that the literature review has limited discussion of specific measures of NQP is critical. We have added a theoretical discussion of digital technologies and specific measures of NQP in Section 3 of the article to strengthen the conceptual foundation. The level 1 indicator adopts intangible labor data to reflect the intensity of the port's investment in intangible assets such as patents, software, brands, etc., which is the core characterization of innovation-driven productivity; the fixed labor data directly reflects the comprehensive processing capacity of the port logistics system, which also affects the process of automation and intelligent transformation; high-quality talents are the human capital basis for promoting digital transformation and technological innovation; the financial risk indicator reflects the enterprise's investment The financial risk indicator reflects the enterprise's investment capacity, which affects the introduction of new technologies and facility upgrading; the future development indicator reflects the agility of the port logistics system and its ability to optimize the operation of the existing assets, which is highly compatible with the cost reduction and efficiency enhancement goals required by the new quality productivity. (See lines 30 to 32 on page 8 and lines 8 to 9 on page 20 for details)

**Q2: This manuscript employs multiple regression models, such as instrumental variable regression and fixed-effects regression. However, it lacks a detailed discussion on the economic rationale for choosing these models and their applicability. Supplementing relevant econometric methodological justifications is suggested.**

**Response:** In response to your comments, we have included a rationale for selecting the baseline regression analysis model and a discussion of model fit in Section 3.3, which details the econometric methodological rationale. This study adopts a baseline regression analysis model, which can effectively control individual heterogeneity, such as port location advantage and policy additions, to avoid estimation errors caused by omitted variables. There may be a bidirectional causal relationship between digital technology and new quality productivity of port logistics, and the use of a fixed-effects model can eliminate individual effects that do not change over time and alleviate some endogenous disturbances through demeaning treatment. (See lines 13 to 14 on page 13 and lines 1 to 4 on page 14 for details)

**Q3: This manuscript uses telephone penetration as an instrumental variable, but it lacks a detailed discussion on whether this variable satisfies the relevance and exogeneity assumptions.**

**Response:** In the endogeneity test, we re-selected the breadth of digital technology application as an instrumental variable, referring to Yang Peng's approach. Yang Peng used text analysis to search, match, and count the word frequency of keywords related to “digital technology” in the annual reports of listed companies according to the breadth of digital technology application, and expanded the sample in the year of study. We used established econometric techniques to re-evaluate the methodology and enhance the empirical rigor of the study. (See lines 4 to 24 on page 16 for details)

**Q4: The current model illustrations are relatively simplistic. It is recommended to include more intuitive causal pathway diagrams, such as how digital technology influences new-quality productivity through infrastructure and the digital economy. Adding more details would help readers better understand the research framework.**

**Response:** Thank you for your valuable suggestion. We have revised Figure 1 by adding two mediating variables in the new quality productivity of port logistics enabled by digital technology to make it consistent with the theoretical framework; meanwhile, we have added a new structural equation modeling diagram for the mechanism test analysis in Chapter 4.5, which presents in detail the composition of the evaluation indicators of digital technology and new quality productivity, as well as the influence coefficients among the explanatory variables, the explanatory variables and the mediating variables. (See lines 30 to 32 on page 8 and lines 8 to 9 on page 20 for details)

**Q5: This manuscript selects three major ports—Ningbo, Shanghai, and Tangshan—as research objects, which are somewhat representative. However, further discussion is needed on whether the findings can be generalized to all ports in China or other logistics sectors. It is suggested to add explanations regarding the limitations of the dataset.**

**Response:** Your discussion of the generalizability of the study findings and the limitations of the dataset is extremely valuable. Due to research resources and time constraints, a more in-depth discussion of dataset limitations is not possible at this time. In subsequent studies, we will further expand the scope of the study and refine our analysis of this issue.

**Q6: This manuscript cites a large number of Chinese references, but the formatting is inconsistent. For example, references [18], [19], and [27] are formatted differently. It is recommended to standardize the reference formatting for consistency.**

**Response:** We have overhauled the citation formatting to ensure that the citation formatting and the formatting of the reference list are consistent throughout the manuscript. [18] Fan S. Influencing factors and countermeasures on intelligent transformation and upgrading of logistics firms: A case study in China[J]. Plos one, 2024, 19(4): e0297663. [19] Burinskiene A, Daskevic D. The investigation on the application of digital technologies for logistics business competitiveness[J]. Tehnički glasnik, 2024, 18(4): 626-637. [27] Zhao Huida, Liu Jiaguo, Hu Xiyuan. Servitization with blockchain in the maritime supply chain[J]. Ocean & Coastal Management, 2022, 225: 106195.( See lines on page 23 to page 26 for details)

Thank you again for your positive and constructive comments and suggestions on our manuscript.

**Reviewer3:**

**Q1: What is the innovative contribution of this paper?**

**Response:** Thank you very much for your request to clarify the innovative contribution, which helps readers better understand the research value. This paper breaks through the traditional technological instrumentalism perspective, constructs the theoretical framework of "digital technology penetration - new digital infrastructure empowerment - technology application internalization - productivity transformation", and systematically reveals the intrinsic mechanism of digital technology influencing the new quality of port logistics. This paper breaks through the traditional technological instrumental perspective to construct the theoretical framework of "digital technology penetration - new digital infrastructure empowerment - technology application internalization - productivity transformation", which systematically reveals the intrinsic mechanism of digital technology affecting the new quality of productivity in port logistics. (See lines 5 to 15 on page 3 and lines 1 to 15 on page 4 for details)

**Q2: The sample size is too small, and the data granularity is too coarse.**

**Response:** Your point about small sample sizes and coarse data granularity is spot on. We have expanded the dataset to cover the period from 2013 to 2023, and added data from 2023 to try to improve the data situation. However, due to the specificity and complexity of port data acquisition, it is difficult to further refine the granularity of the data in the short term, and we will continue to pay attention to and improve the data in our subsequent studies. (See lines 19 to 23 on page 14 and line 1 on page 15 for details)

**Q3: The metrics are not clearly defined. For example: What constitutes "New-Quality Productivity"? How is "Digital Technology" measured?**

**Response:** We add a discussion of specific measures of digital technology and new quality productivity in Section 3 of the article to clearly define the relevant indicators. The three-dimensional level 1 indicators cover the whole chain of “resources-connections-applications”, with digital informatization indicating the reserve of digital assets (supply side), network interconnectivity measuring the connectivity (circulation side), and digitization of facilities reflecting the effectiveness of applications (demand side). The number of domain names used in the secondary indicators reflects the activity of digital identity of regional economic subjects, which is the core characterization of digital assets precipitation; the scale of international Internet access users determines the efficiency of cross-border data circulation, which is a key indicator for building an open digital hub; the cell phone penetration rate can reflect the fairness of digital access in the society in one aspect, and provide the underlying technical support for the Internet of Things, mobile payment and other scenarios; the density of fiber optic users has a direct impact on cloud computing and application of port logistics. The density of fiber optic users directly affects the quality of development of new types of business such as cloud computing, telecommuting and online learning in port logistics; the digitalization results reflect the depth of integration of digital technology and physical facilities; the digital transformation index is constructed on the basis of composite indicators such as the enterprise cloud rate and the access rate of the industrial Internet platform, reflecting the maturity of the digital ecosystem of the city. (See lines 30 to 32 on page 8 and lines 8 to 9 on page 20 for details)

Thank you again for your positive and constructive comments and suggestions on our manuscript.

We tried our best to improve the manuscript and made some other changes in the manuscript. These changes will not influence the content and framework of the paper. Here we did not list the changes but marked the changed parts in red in the revised version.

Once again, we sincerely thank the reviewers for their valuable comments, which are of great significance in improving the quality of this paper. If you have any other questions or suggestions about the revision, please feel free to point them out, and we will continue to improve it.
