# Supplementary material for: Research on the impact mechanism of digital technology on the new-quality productivity of port logistics
Source: PLoS One. 2025 Sep 26;20(9):e0333071. doi: 10.1371/journal.pone.0333071 (PMC12469090; doi:10.1371/journal.pone.0333071)
Supplement: Attachment — Submitted filename: 0802_Response to Reviewers.docx [file pone.0333071.s002.docx]

Response Letter

Dear Editor and Reviewers:

Thank you very much for your valuable feedback on our manuscript entitled “Research on the Impact Mechanism of Digital Technology on the New-Quality Productivity of Port Logistics”. (Manuscript ID: PONE-D-25-08375). These feedbacks provide important guidelines for improving the quality of the article, and we have carefully sorted out and actively implemented the modifications, and now report the details as follows:

**Reviewer1:**

**Q1: The innovation need to be improved.**

**Response:** To enhance the innovation of the article, we have made revisions in several aspects: In terms of research topic selection, the introduction emphasizes the lack of mechanistic exploration in existing studies on the "systematic driving effect of digital technology on new-quality productivity in port logistics". (See lines 4 to 6 on page 4 for details) We innovatively take "digital technology" and "new-quality productivity in port logistics" as core interrelated objects, focusing on their intrinsic action logic. (See lines 3 to 5 on page 17 for details) In terms of research methods, we supplemented the description of the explained variable by clarifying that the evaluation index of new-quality productivity in port logistics is constructed from multiple dimensions, which improves the scientificity of variable measurement. Additionally, a two-way causality test was added in the endogeneity test to further verify the robustness of the conclusions. (See line 1 on page 25 to line 1 on page 26 for details)

**Q2: Standardize citation formats throughout the manuscript and ensure consistency in reference list formatting.**

**Response:** As requested, we have thoroughly standardized the citation formats throughout the entire manuscript and ensured strict consistency in the reference list. Specifically, the revisions include: in the manuscript, the formatting of authors' names has been kept consistent. In the references section, the formatting of authors' names has been standardized, and publication information such as journal volume, issue, and page ranges has been uniformly presented, with punctuation and spacing ensured to be standardized and consistent. (See line 1 on page 36 to line 10 on page 40 for details)

**Q3: Please adjust the table formats to ensure that the tables are clearly presented.**

**Response:** Thank you for your feedback on the table formatting. We have carefully adjusted the formatting of all tables in the manuscript to enhance clarity. Specifically, we have standardized the alignment of columns, optimized border lines, and ensured consistent spacing between cells and text, thereby improving readability. These revisions aim to make the tables easier to understand while being consistent with the overall layout of the manuscript. (See table 1 on page 16 and 17, table 2 on page 18 and 19, table 3 on page 21, table 4 on page 22 and 23, table 5 on page 24 and 25, table 6 on page 25 and 26 for details)

We tried our best to improve the manuscript and made some other changes in the manuscript. Here we did not list the changes but marked the changed parts in red in the revised version.

Once again, we sincerely thank the reviewers for their valuable comments, which are of great significance in improving the quality of this paper. If you have any other questions or suggestions about the revision, please feel free to point them out, and we will continue to improve it.
